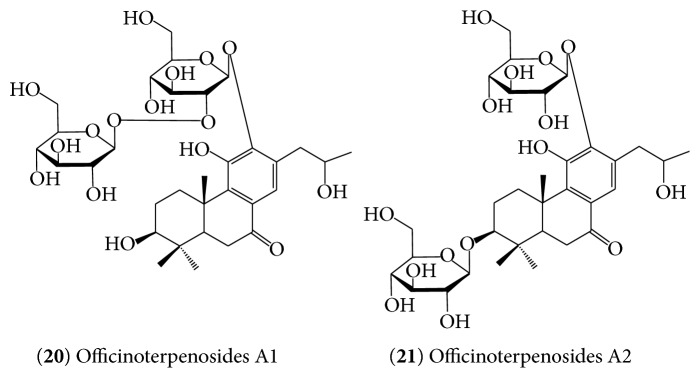# The Therapeutic Potential of Rosemary (*Rosmarinus officinalis*) Diterpenes for Alzheimer's Disease

**DOI:** 10.1155/2016/2680409

**Published:** 2016-01-28

**Authors:** Solomon Habtemariam

**Affiliations:** Pharmacognosy Research Laboratories & Herbal Analysis Services UK, Chatham-Maritime, Kent ME4 4TB, UK

## Abstract

Rosemary (*Rosmarinus officinalis* L.) is one of the most economically important species of the family Lamiaceae. Native to the Mediterranean region, the plant is now widely distributed all over the world mainly due to its culinary, medicinal, and commercial uses including in the fragrance and food industries. Among the most important group of compounds isolated from the plant are the abietane-type phenolic diterpenes that account for most of the antioxidant and many pharmacological activities of the plant. Rosemary diterpenes have also been shown in recent years to inhibit neuronal cell death induced by a variety of agents both* in vitro* and* in vivo*. The therapeutic potential of these compounds for Alzheimer's disease (AD) is reviewed in this communication by giving special attention to the chemistry of the compounds along with the various pharmacological targets of the disease. The multifunctional nature of the compounds from the general antioxidant-mediated neuronal protection to other specific mechanisms including brain inflammation and amyloid beta (A*β*) formation, polymerisation, and pathologies is discussed.

## 1. Introduction

*Rosmarinus officinalis* L. (family, Lamiaceae), commonly known as rosemary, is one of the most popular perennial culinary herbs cultivated all over the world. Both fresh and dried leaves of rosemary have been used for their characteristic aroma in food cooking or consumed in small amount as herbal tea, while rosemary extracts are routinely employed as natural antioxidant to improve the shelf life of perishable foods. In the latter case, the European Union has approved rosemary extract (E392) as a safe and effective natural antioxidant for food preservation [1]. The plant is also known to be employed in traditional medicines in many countries even far beyond its native Mediterranean region where it grows wild. Among the pharmacologically validated medicinal uses of rosemary are antibacterial [2], anticancer [3, 4], antidiabetic [5], anti-inflammatory and antinociceptive [6–8], antioxidant [5, 9], antithrombotic [10], antiulcerogenic [11, 12], improving cognitive deficits [13], antidiuretic [14], and hepatoprotective [15, 16] effects. The other major use of rosemary is in the perfumery industry where the essential oils are employed as natural ingredients of fragrances.

The culinary, medicinal, and fragrance uses of rosemary are attributed to the vast arrays of chemical constituents collectively known as plant secondary metabolites. Of these, one group are small molecular weight aromatic compounds called essential oils which play vital role in the fragrance and culinary properties of the plant. Essential oils of rosemary dominated by 1,8-cineole, *α*-pinene, camphene, *α*-terpineol, and borneol as principal constituents [17, 18] are also responsible for various pharmacological effects of the general antioxidant [8] and antimicrobial [2, 19–25] properties known for many essential oils, as well as other effects including anticarcinogenic activities [26]. The other group of secondary metabolites of rosemary are polyphenolic compounds including the flavonoids (e.g., homoplantaginin, cirsimaritin, genkwanin, gallocatechin, nepetrin, hesperidin, and luteolin derivatives) and phenolic acid derivatives (e.g., rosmarinic acid) [27–29]. By far the most important group of rosemary compounds that gain significant attention in recent years, however, are the unique class of polyphenolic diterpenes. In this review, the chemistry and pharmacology of rosemary diterpenes are scrutinised by giving special emphasis to their therapeutic potential for Alzheimer's disease (AD).

Accounting for an estimated 60 to 80 percent of dementia cases in the elderly populations, AD has become one of the major global health challenges of the century. The worldwide prevalence of dementia is now estimated to exceed 36 million cases with a further projection of 115 million by 2050 [30–32]. One of the current well-accepted pathologies of AD is the “amyloid hypothesis” that puts the accumulation and aggregation of amyloid-beta (A*β*) as the major cause of the progressive neuronal cells deaths in the brain. Neuronal deletion particularly in the cortex region is now known to lead to cognitive impairment including acquired learning skills and memory. The hosts of behavioural symptoms arising from AD include agnosia, aphasia, apraxia, erratic emotion, sleep disorders, and interpersonal/social deterioration [33, 34]. Numerous studies have shown that these clinical symptoms of AD are associated with the loss of cholinergic neurons induced by toxicants such as A*β*, reactive oxygen species (ROS), inflammatory cascades, and excitotoxicity mechanisms. Critical to the AD pathology is the basal forebrain region from where cortical cholinergic neurons originate. The loss of neurons in the basal forebrain has been shown to correlate with the degree and severity of clinical symptoms of AD [34]. To date, the handful of drugs available to treat AD are the acetyl cholinesterase (ACHE) inhibitors (e.g., rivastigmine, galantamine, tacrine, and donepezil) and N-methyl-D-aspartate (NMDA) receptor antagonist (memantine) which have some benefit in alleviating the clinical symptoms of AD [35]. Drug of cure for AD is, however, neither available nor within sight, and the search of new drugs from natural sources should be considered as a viable strategy for the future control of the disease. One group of compounds of interest are the rosemary diterpenes which are comprehensively assessed in this communication for their therapeutic potential for AD. Special emphasis is given to the structural features of the compounds with respect to their effects against specific AD target.

## 2. Overview of Rosemary Diterpenes

### 2.1. Biosynthetic Perspective

**Figure 1:**
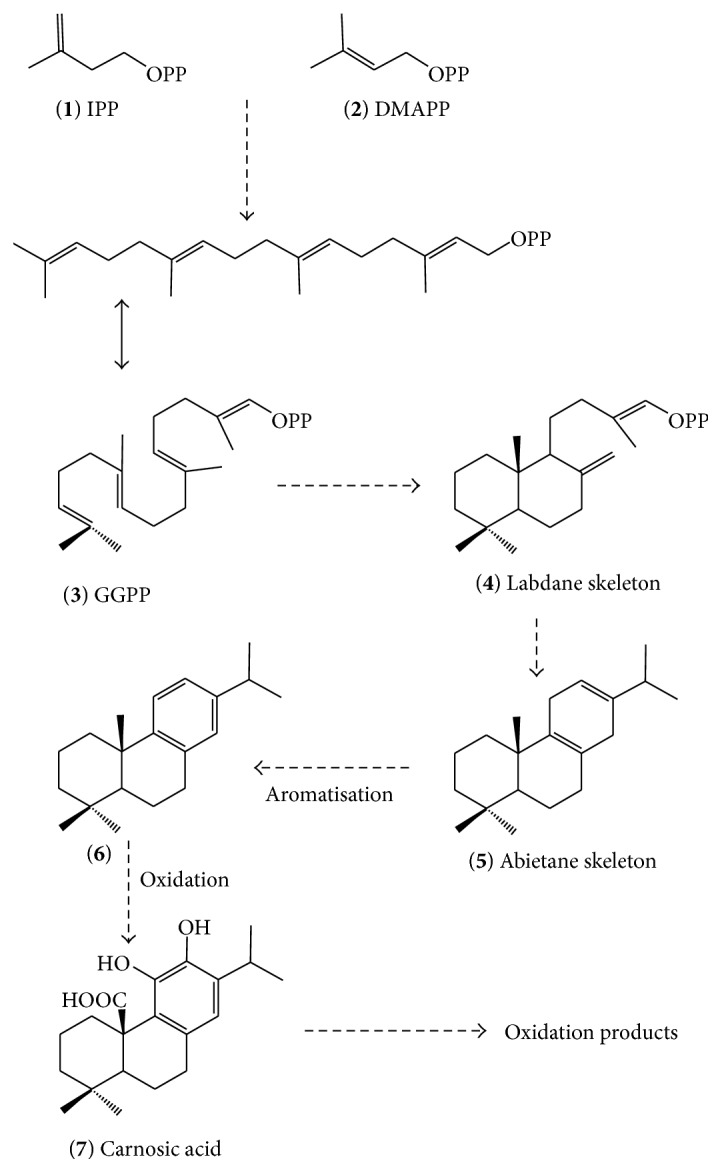
Schematic presentation of the biosynthetic pathway of rosemary diterpenes.

Biosynthetically, diterpenes are derived from the terpenoids or mevalonate pathway and hence composed of repeating 5-carbon backbone skeleton, isoprene unit(s). The two known isoprene building blocks are isopentenyl pyrophosphate (IPP,** 1**, Figure 1) and dimethylallyl pyrophosphate (DMAPP,** 2**) that polymerises in head-to-tail fashion to form the 20-carbon diterpene precursor (4 isoprene units) called geranylgeranyl pyrophosphate (GGPP,** 3**). The processing of the GGPP through reactions including cyclization, aromatisation, rearrangements, and a series of reaction steps emanating from the loss of the phosphate group, including removal of the carbonium ion, results in the formation of the diterpene subgroups. The class of diterpenes in rosemary identified so far is the abietane type (**5**–**7**) which is composed of six–membered tricyclic ring system of which one is aromatic (e.g.,** 7**) [36]. Biosynthetically, abietane-type diterpenes are known to derive from their immediate precursor, labdane subclass (**4**), as shown in Figure 1. The labdane group of diterpenes on their own are diverse natural products that have been shown to include compounds of novel structural and biological significances [37–40].

### 2.2. Diversity of Rosemary Diterpenes

**Figure 2:**
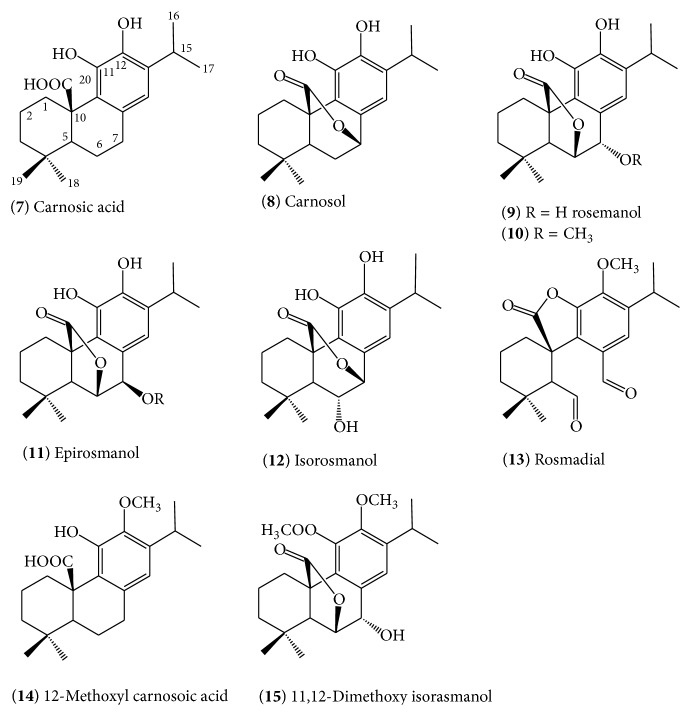
Carnosic acid and related abietane-type diterpenes of rosemary.

The various types of diterpenes isolated from rosemary are shown in Figure 2. The basic skeleton of all of these diterpenes in rosemary appears to be carnosic acid (**7**) which was first isolated from the plant by Wenkert et al. [41] in 1965. It is now well known that this compound is the major constituent of rosemary that accounts to 1.5–2.5% of the dried leaves though even higher amounts have been reported [41]. Like many other secondary metabolites, the concentration of carnosic acid (**7**) and other diterpenes in rosemary could vary due to a host of environmental factors (e.g., sun light intensity and water stress) and growth conditions [42–46] as well as genetic factors as there are now several varieties that could yield the compound in up to 10% yield by dry weight [47]. Carnosic acid (**7**) is not unique to rosemary and its distribution in sage and other taxonomically related species has been revived recently by Birtić et al. [48]. Other taxonomically unrelated plants such as* Premna species* have also shown to synthesise pharmacologically significant abietane-type diterpenoids with even more aromatisation than those shown for rosemary diterpenoids in Figure 2 [49].

Although carnosic acid (**7**) is the principal constituent of rosemary extracts, it is not a very stable compound once extracted and may undergo oxidation to form the *γ*-lactone diterpene, carnosol (**8**). In fact, the conversion of (**7**) to (**8**) in extracts of* R. officinalis* and* Salvia officinalis* has been well documented [50], and the latter was considered as the principal constituent of the plant in earlier studies. In addition to carnosol (**8**), the oxidation of (**7**) is also known to yield rosmanol (**9**) which differs from carnosol by possessing a free hydroxyl group at C-7 position and the *γ*-lactone formed* via* the C-20-C-6 route [50–53]. The epimeric form of rosmanol with stereochemistry difference at C-7 position has also been demonstrated by the identification of (**11**) (epirosmanol [54]). An enzyme catalysed conversion of carnosic acid (**7**) to lactone derivatives* via* singlet oxygen-mediated reactions has been suggested as a possible mechanism of these diterpene lactones formation [55–57]. Enzymatic dehydrogenation and free radical attack are now also generally considered as a common route for the formation of various oxidation products of (**7**) [55, 58]. An alternative structure, isorosmanol (**12**) [57], where the lactone ring is formed* via* the C-6 instead of the C-7 hydroxyl position, has also been identified in rosemary extract. The further route of structural diversification in rosemary diterpenes comes through methoxylation and hence the 12-methoxyl derivative of carnosic acid (**14**) and 11,12-dimethoxy isorosmanol (**15**) have been identified. Methoxylation at the 7-position is also evident as 7-methoxy-rosmanol (**10**) has been identified from rosemary [51]. All these diterpenes are relatively polar and are not found in the essential oil of rosemary [59].

**Figure 3:**
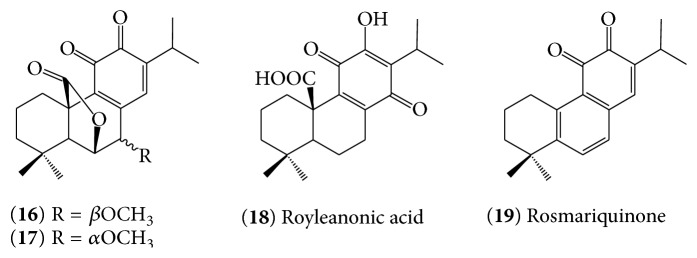


The other structurally interesting group of rosemary diterpene derivatives are diterpene quinones (**16**)–(**19**) (Figure 3). Mahmoud et al. [60] reported the isolation and structural elucidation of two new abietane-type diterpenoid* O*-quinones, rosmaquinone A (**16**) and rosmaquinone B, (**17**) along with another known diterpene quinone, royleanonic acid (**18**) and rosmanol. Another example of diterpene quinone identified from rosemary was rosmariquinone (**19**) [61].

Glycosylation is the common route of structural diversification in natural products. The study by Zhang et al. [54] has resulted in the identification of polar diterpene glycosides named as officinoterpenosides A1 (**20**) and A2 (**21**) (Figure 4). These polar compounds also differ from the carnosic acid derivatives (**7**–**15**) not only by their glycosylation and different oxygenation pattern but also by having an altered side chain whereby the 16-methyl group has migrated to the C17 position.

Munné-Bosch and Alegre [44] have analysed the relative concentrations of diterpenes in rosemary tissues. In general, the level of carnosic acid (**7**) was about 6-fold higher than other derivatives such as 12-*O*-methylcarnosic acid (**14**) and carnosol (**8**), which (the latter two) were found in similar concentrations. On the other hand, isorosmanol (**11**) was found at slightly lower concentrations than carnosol (**8**) while the 11,12-di-*O*-methylisorosmanol (**15**) was about 10 times less abundant than isorosmanol (**11**). The rosmanol (**9**) concentration is regarded as a trace amount [44]. The most important diterpenes in terms of biological significance of the rosemary however remain to be carnosic acid (**7**) and carnosol (**8**) which are most abundant (~5% the dry weight) and shown to account for over 90% of rosemary's antioxidant effects [48, 62]. Dried rosemary could contain about 0.2–1% carnosol (**8**) [63] while many commercially available extracts may be optimised to contain approximately 10.3% carnosol (**8**) [64].

*Bioavailability*. Doolaege et al. [65] have studied the absorption, distribution, and elimination of carnosic acid (**7**) in rats following administration* via* the intravenous (20.5 ± 4.2 mg/kg) and oral (64.3 ± 5.8 mg/kg) routes. Their study revealed that the bioavailability of (**7**) after 360 min following the intravenous dosage was 40.1%. The study also showed that traces of (**7**) were found in various organs in its free form while elimination in the faeces after 24 h after oral administration was 15.6 ± 8.2% [65]. Another study by Vaquero et al. [66] emphasised on the oral route of (**7**) where the glucuronide conjugates were found to be the main metabolites detected in the gut, liver, and plasma. The other metabolites identified were the 12-methyl ether and 5,6,7,10-tetrahydro-7-hydroxyrosmariquinone of (**7**) [66]. Since these metabolites were detected as early as 25 min following oral administration, it was reasonable to conclude that rosemary diterpenes are bioavailable. Interestingly, the free form of (**7**) as well as its metabolites was detected in the brain [66] suggesting possible effect in this vital organ.

## 3. Pharmacological Targets of Rosemary Diterpenes Related to AD Therapy

### 3.1. General Pharmacological Effect of Rosemary Diterpenes on the Brain and Memory

In an attempt to investigate the effect of rosemary tea consumption on brain function, Ferlemi et al. [67] have recently tested the potential anxiolytic- and antidepressant-like behaviour effect on adult male mice. The result showed that oral intake of rosemary tea for 4 weeks has shown a positive effect without altering memory/learning when assessed by passive avoidance, elevated plus maze and forced swimming tests. In an olfactory bulbectomy procedure in mice, MacHado et al. [68] have also demonstrated that rosemary extract possesses antidepressant-like effect and is also able to abolish ACHE alterations although the spatial learning deficit induced by the procedure was not altered. Carnosic acid (**7**) has also shown to have neuroprotective effects on cyanide-induced brain damage in cultured rodent and human-induced pluripotent stem cell-derived neurons* in vitro* and* in vivo* in various brain areas of a non-Swiss albino mouse model [69]. As discussed in the later sections, this effect is likely to be mediated* via* upregulation of transcriptional pathways related to antioxidant and anti-inflammatory mechanisms [69]. Protective effects of carnosol (**8**) on rotenone-induced neurotoxicity in cultured dopaminergic cells were also observed* in vitro* in parallel with downregulation of apoptotic mechanisms [70]. It is also worth noting that other components of rosemary, such as essential oil constituents, are known to alter brain function at therapeutic doses. For example, the cognitive enhancing power of rosemary component, 1,8-cineole, has been well documented [71]. In agreement with these observed effects of the isolated compounds (**7**,** 8**), the crude extract of rosemary has been shown to improve memory impairment when tested* in vivo* using the scopolamine-induced dementia model of AD [72].

### 3.2. Antioxidant Mechanisms

A number of simple* in vitro* experiments where the antioxidant potential of rosemary diterpenes is demonstrated include lipid peroxidation and protection of cells from oxidative cell death [73, 74]. Readers must however bear in mind that the antioxidant potential of rosemary extracts and diterpenes on food preservation and various biological models have been established up to the level of large-scale commercial exploitations. The emphasis in this communication is therefore limited to highlighting mechanisms relevant to neurodegenerative diseases. In this respect, Hou et al. [75] have shown that carnosic acid (**7**) protects neuronal cells from ischemic injury by scavenging ROS. The antioxidant mechanisms of (**7**) and carnosol (**8**) are dependent on the loss of hydrogen from their phenolic hydroxyl groups leading to formation of quinone derivatives [76, 77]. Through this antioxidant mechanism, (**7**) can protect neuronal cells from oxidative damage both* in vitro* and* in vivo*. Numerous reports during the last few decades including ours have shown that the antioxidant mechanism and/or radical scavenging effect of polyphenolic natural products is exceptionally prominent when the compounds possess the catechol functional group [78–89]. The formation of the various diterpene derivatives as the oxidation products of (**7**) is also inherently related to its ability to interact with ROS [50, 90].

The induction of phase II detoxifying enzymes is an important defence mechanism for the removal of xenobiotics and other toxicants of internal and external origin. A large body of evidence to date indicates that the erythroid derived 2-related factor 2 (Nrf-2) is involved in the antioxidant response elements- (AREs-) mediated induction of genes for a variety of antioxidant enzymes, including phase II detoxifying enzymes [91–93]. The expression of many thiol-regulating enzymes, such as glutathione S-transferase, glutamylcysteine synthetase, and thioredoxin reductase, has also shown to be dependent on Nrf-2 [94]. Of the various mechanisms described for these antioxidant effects is direct S-alkylation of the cysteine thiol of the Kelch-like ECH-associated protein 1 (Keap1) protein by the “electrophilic” quinone derivative of (**7**) [95]. Keap1 is a regulatory protein associated with the transcriptional factor Nrf2 that binds to the ARE [96]. The binding of electrophiles compounds with the cysteine residues on Keap1 protein and the subsequent S-alkyl adduct formation will allow the migration of the Nrf2 to the nucleus. Nrf2 can then promote genes expression by binding to AREs of phase II genes. Through this mechanism, the application of electrophile compounds as antioxidant and neuroprotective agents has been well documented in the various literature [95, 97–99].

Carnosol (**8**) possesses high electrophilic activity and has been reported to activate Nrf2, phase II detoxifying enzyme genes, and antioxidant enzymes [99, 100]. Direct interaction of (**8**) with cysteine residues of the nuclear factor kappa B (NF-*κ*B) has also been demonstrated [101–103]. In a similar manor, carnosic acid (**7**) has been shown to protect neuronal HT22 cells through activation of the antioxidant-responsive element [104]. The free carboxylic acid and catechol hydroxyl moieties have been shown to play critical role in these effects [104]. All the available evidence now therefore suggests that the major rosemary constituents (**7** and** 8**) protect neurons against oxidative stress by activating the Keap1/Nrf2 pathway. Xiang et al. [104], for example, have demonstrated that (**7**) and (**8**) could protect HT22 cells against oxidative glutamate toxicity through mechanisms involving activation of the transcriptional ARE of phase II genes including heme oxygenase-1, NADPH-dependent quinone oxidoreductase, and *γ*-glutamyl cysteine ligase, all of which provide neuroprotection by regulating the cellular redox system. Through antioxidant mechanism, (**7**) does also protect the lipopolysaccharide- (LPS-) induced liver injury through enhancement of the body's cellular antioxidant defence system as the levels of superoxide dismutase, glutathione peroxidase, and glutathione in serum and liver after the LPS challenge were restored [105]. Pretreatments of RAW264.7 macrophages with (**7**) also resulted in a significant reduction of the hydrogen peroxide- or LPS-induced generation of ROS and nitric oxide while the heme oxygenase-1 (HO-1) protein expression was time- and dose-dependently upregulated [76]. Moreover, carnosol (**8**) has been shown to enhance the glutathione S-transferase (GST) and quinone reductase activity* in vivo* [105].

The therapeutic potential of rosemary diterpenes for AD must be seen in conjunction with the role of oxidant-antioxidant mechanisms in the pathology of the disease. A number of studies have clearly outlined the direct association between ROS-mediated macromolecular cell damage and neuronal cell death in AD, particularly in brain regions where A*β* is highly prevalent [106, 107]. Interestingly, neuronal cells in the brain appear to be more susceptible to ROS-mediated cell damage than any other cell types for numerous reasons including high oxygen consumption [108], high level of polyunsaturated fatty acids content of cell membrane [109], association of the NMDA receptor activation with ROS-induced neuronal apoptosis [110], and poor level of antioxidant defences including the catalase, glutathione peroxidase, and vitamin E contents [111]. Furthermore, antioxidant defences in AD have been found to be highly suppressed as low level of SOD [112] and reduced form of glutathione (GSH) [113, 114] as well as mitochondrial dysfunction [115] are all common features of AD. Hence, the numerous reports on the antioxidant effects of rosemary diterpenes along with their specific effect on neuronal cells through the abovementioned antioxidant mechanisms imply that they should be considered for further development as anti-Alzheimer's agents.

*Metal Chelation*. High level of metal ions such as copper, zinc, and iron have been found in the amyloid plaques of AD brains [116–118]. Higher millimolar level of unregulated metal ions in the brain has also been shown to arise due to age related deterioration of the blood-brain-barrier leading to unchecked access of the brain to metal ions [119]. As described in the later section, these metal ions play critical role in A*β*-induced neurotoxicity in AD. Hence, a potent metal chelative effect of a drug is an important feature of anti-AD therapy. Our own study on polyphenolic compounds in the last two decades has revealed that their biological effect including enzyme inhibition could be partly explained by their ability to chelate iron and other redox metals and, for such effect, one of the best structural features in a molecule is the orthodihydroxyl functional moiety [78–89].

The structural features of (**7**) and (**8**) are in favour of strong metal chelation properties. Carnosol (**8**) has been shown to inhibit Cu^2+^-induced LDL oxidation [120] but, most importantly, metal (e.g., iron) chelation is one of the known mechanisms of antioxidant effects. Furthermore, iron absorption from the gut is strongly suppressed by rosemary extract [121].

### 3.3. Anti-Inflammatory Mechanisms

The roles of Nrf2 and the antioxidant protein HO-1 in neuroinflammatory response have been well established. The search for effective Nrf2/HO-1 activators that modulate the microglia inflammatory response in AD would therefore have significant therapeutic value. A recent study has further revealed that Nrf2 activation inhibits inflammatory gene expression [122] through mechanisms involving HO-1 [123]. Lian et al. [124] have also shown that carnosol (**8**) and rosemary essential oils inhibit the adhesion of tumour necrosis factor-*α*- (TNF-*α*-) induced monocytes to endothelial cells and suppress the expression of intercellular adhesion molecule (ICAM-1) at the transcriptional level* in vitro*. The anti-inflammatory effect of (**8**)* via* inhibition of the TNF-*α*-induced protein expression of ICAM-1 was also shown to be extended to other cell surface molecules such as the vascular cell adhesion molecule- (VCAM-) 1 and E-selectin in endothelial cells as well as interleukin- (IL-) 8 and the monocyte chemoattractant protein- (MCP-) 1 [125]. Moreover, Foresti et al. [126] have shown that (**8**) inhibits the TNF-*α*-induced signaling pathways through inhibition of inhibitor of nuclear factor kappa-B (IKK-*β*) activity as well as the upregulation of HO-1 expression. At the concentration of 5–20 *μ*M, (**8**) was demonstrated to upregulate Nrf2 and HO-1 leading to downregulation of the inflammatory response (TNF-*α*, prostaglandin E-2, and nitrite) [126]. Carnosic acid (**7**) was similarly shown to inhibit the expression of cytokine-induced adhesion molecules on endothelial cells surface leading to inhibition of monocyte-cell adhesions [127]. It does also potently inhibit the LPS-induced rise in serum levels of the proinflammatory cytokines (TNF-*α* and IL-6)* in vivo* [128]. Both (**7**) and (**8**) have also shown to inhibit the phorbol 12-myristate 13-acetate- (PMA-) induced ear inflammation in mice with EC_50_ of 10.20 *μ*g/cm^2^ and 10.70 *μ*g/cm^2^, respectively. This activity was coupled with reduced level of expression of IL-1*β* and TNF-*α* and cyclooxygenase-2 (COX-2). In another study [129], both (**7**) and (**8**) inhibited the formation of proinflammatory leukotrienes in cells with IC_50_ of 7–20 *μ*M as well as purified recombinant 5-lipoxygenase (IC_50_ = 0.1–1 *μ*M). The study also showed that both (**7**) and (**8**) potently antagonise intracellular Ca^2+^ mobilisation induced by a chemotactic stimulus, coupled with inhibition of ROS generation [129]. The LPS-induced nitric oxide production in Raw 264.7 cells was also shown to be inhibited by (**8**) with IC_50_ of 9.4 *μ*M [130]. In an* in vitro* model of brain inflammation, (**7**) inhibited the LPS-induced activation of cells of the mouse microglial cell line MG6 [131], releasing inflammatory cytokines such as IL-1*β* and IL-6. The nitric oxide production associated with a decrease in the level of inducible nitric oxide synthase has also been reported for (**7**) [131].

Glial cells are the major inflammatory cells of the brain which produce massive amount of proinflammatory cytokines (e.g., IL-1*β*, IL-6, and TNF-*α*) upon activation. Numerous studies have highlighted the fact that high levels of these inflammatory cytokines are critical in the coordination of brain inflammation in AD [132, 133]. Moreover, both microglia and astrocytes have been shown to be highly regulated in AD brains [133, 134]. The potent anti-inflammatory activity of rosemary diterpenes in both the microglial cells [126, 135] and other inflammatory models therefore suggests their potential in tackling AD.

### 3.4. A*β* Mechanisms

Generally, amyloid plaques and neurofibrillary tangles (NFT), which are closely linked to the formation of toxic insoluble aggregates of A*β*, have shown to be the two most common pathological hallmarks of AD [136–138]. The A*β* is formed from the neuronal transmembrane glycoprotein (100–130 kDa) called the amyloid precursor protein (APP). The *α*-, *β*-, and *γ*-secretases are the three major proteolytic enzymes that process APP [139] through two major pathways: the amyloidogenic and nonamyloidogenic pathways. The non-amyloidogenic-dependent pathway involves APP processing through *α*-secretase leading to the generation of nonpathogenic amyloid products. In the amyloidogenic pathway, *β*-secretase processes APP at the N-terminus of the A*β* domain to generate the membrane-attached fragment, C99, and the sAPP*β* fragment [140]. Further cleavage of the C99 fragment by *γ*-secretase leads to the formation of the two most common forms of A*β* peptides, A*β*1–40 (90%) and A*β*1–42 (10%), along with other fragments. To date, a number of therapeutic agents that inhibit APP processing have been identified and some appear to be in clinical trials [141]. Of these, inhibitors of *β*-secretase 1 (BACE1) appear to be most important as this enzyme takes the first rate limiting step in APP processing [142]. To the best of the author's knowledge, an inhibitory effect of rosemary diterpenes on *β*-secretase activity has not been demonstrated but a promising effect on *α*-secretase has been reported by Meng et al. [143]. In their study using the SH-SY5Y human neuroblastoma cells, carnosic acid (**7**) showed 61% suppression of A*β*42 secretion when tested at the concentration of 30 *μ*M. The effect was also coupled with enhanced mRNA expressions of *α*-secretase but not the *β*-secretase BACE1. Hence, the mechanism of action of (**7**) for APP processing inhibition appears to be through promotion of the normal non-amyloidogenic-dependent pathway. Similar results were also demonstrated by Yoshida et al. [144] where A*β* peptides (1–40, 1–42, and 1–43) production in U373MG human astrocytoma cells was suppressed by (**7**) (50 *μ*M). The study also revealed a 55 to 71% inhibition of A*β* release coupled with effect on mRNA expressions of an *α*-secretase, but once again not the *β*-secretase BACE1 [144].

Once A*β* is formed, it undergoes a serious of polymerisation processes leading to the formation of insoluble precipitates. It has been shown that small soluble oligomers as well as amyloid fibril aggregates induce toxicity to neuronal cells in AD [145–149]. Hence, various classes of natural and synthetic compounds that inhibit the polymerisation and stability of A*β* aggregates can be employed as viable therapeutic agents for AD. Some of these agents identified to date include chrysamine G [150], oligopeptides [151–155], and plant polyphenols such as curcumin, myricetin, morin, quercetin, kaempferol (+)-catechin, (−)-epicatechin, nordihydroguaiaretic acid and tannic acid [156–158], antibiotics (e.g., rifampicin [159]), and aspirin [160]. In this connection, Meng et al. [143] have recently investigated the effect of carnosic acid (**7**) on the viability of cultured SH-SY5Y human neuroblastoma cells challenged by A*β*42 or A*β*43. The cellular deletion in these cells treated with A*β*42 or A*β*43 (monomer, 10 *μ*M each) was reported to be partially reversed by treatment with (**7**) (10 *μ*M). The observed effect was also coupled with reduced level of cellular oligomers of A*β*42 and A*β*43 suggesting inhibition of oligomerisation as the possible mechanism of action [143]. These data were also in agreement with the* in vivo* observation where (**7**) has been demonstrated to show beneficial effect in AD models [161]. Rasoolijazi et al. [162] also provided direct evidence to demonstrate the therapeutic potential of (**7**) for AD by using A*β* toxicity* in vivo*. When A*β* (1–40) was injected into the Ca1 region of the hippocampus of rats, neurodegeneration and cognitive impairment were evident as assessed by the passive avoidance learning and spontaneous alternation behaviour tests. Treatment by (**7**) appears to reverse these A*β* (1–40) mediated changes suggesting the therapeutic potential of this compound for AD [162]. The association between A*β* formation and aggregation with metal ions such as copper has been reviewed in many literatures [163–166]. In agreement with this finding, metal chelators have been shown to decrease Alzheimer A*β* plaques [167]. It is now also known that A*β* is a redox-active peptide that reduces transition metals like Cu^2+^ and Fe^3+^ leading to the generation of ROS [168]. Both the polymerisation and toxicity of A*β* are therefore intimately linked to metal ions and ROS [169]. The polymerisation of A*β* itself is shown to be enhanced when the antioxidant defence is diminished [112, 170]. The multifunctional nature of rosemary diterpenes in metal chelation and ROS scavenging is thus likely to contribute to their effect against A*β* polymerisation and toxicity.

### 3.5. ACHE Activity

The impairment of memory and cognitive power in AD has been shown to be associated with the loss of cholinergic neurons in the cortex [171–174]. Under this circumstance where the acetyl choline (ACH) activity in this region is below the normal level, one approach of therapeutic intervention in AD is to minimise the degradation of ACH by its enzyme, ACHE. Even though such drugs have limitation due to their undesirable side effects, an overall beneficial effect in cognitive improvement and behavioural symptoms have been clinically observed [175]. Szwajgier [176] has studied the effect of carnosic acid (**7**) against ACHE along with 35 other phenolic compounds. Interestingly, CA was identified as the most potent.* In silico* molecular interaction study approach on AChE inhibitors has also resulted in the identification of (**7**) as a potential lead drug candidate [177]. The memory enhancing effect of rosemary extract (200 mg/kg, p.o.) in the scopolamine-induced dementia model of AD has also been shown to be linked with direct effect on ACHE activity [72]. While the mRNA expression of butyrylcholinesterase (BuChE) in the cortex was inhibited, its expression in the hippocampus was enhanced by rosemary extract [72]. These effects on the expression of enzymes however could be mediated through indirect effect* via* other mechanisms.

## 4. General Summary and Conclusion

The industrial scale exploitation of rosemary for food preservation and as natural antioxidant additives is attributed to its phenolic constituents. The predominant phenolic compounds that accounts for such effects as well as the various* in vitro* and* in vivo* pharmacological properties of the plant are the abietane type of diterpenes. Structurally, these groups of compounds are based on the steroidal-like terpenoid skeleton but have added pharmacophore of a phenolic structure. The rosemary diterpenoids of pharmacological relevance are represented by (**7**) and (**8**) where the diorthohydroxyl/catecholic functional group is evident. Through these structural features, these compounds display a vast array of pharmacological effects ranging between antioxidant, metal chelation, and anti-inflammatory properties. These very mechanisms do also appear to be involved in the potential therapeutic effect of the compounds for AD. The further effect of rosemary diterpenes in A*β* formation, aggregation, and toxicity accounts for their additional benefit in tackling AD. Given that AD is a complex disease involving many pathological processes, treatment with multifunctional drugs like those demonstrated by rosemary diterpenes constitutes a viable therapeutic approach. The cascade of neurodegeneration process in AD has lots of similarities with other diseases like Parkinson's disease. Interestingly, some of the rosemary diterpenes such as carnosic acid (**7**) have been shown to have beneficial effect in Parkinson's disease model [178, 179]. It is also worth noting that only (**7**) and (**8**) have been extensively investigated for their possible therapeutic effect related to AD. Other interesting diterpenes including the glycosidic forms could have different bioavailability and therapeutic profile. Further research in this field will therefore provide more evidence on the therapeutic potential of rosemary diterpenes. All the available date to date however suggest that their effect on AD is very promising and further research including clinical trials is well warranted.

## Figures and Tables

**Figure 4:**